# Improving treatment of depression in primary health care: a case study of obstacles to perform a clinical trial designed to implement practice guidelines

**DOI:** 10.1017/S1463423614000243

**Published:** 2014-06-27

**Authors:** Linda Richter-Sundberg, Monica Elisabeth Nyström, Ingvar Krakau, Christer Sandahl

**Affiliations:** 1 PhD Student, Department of Public Health and Clinical Medicine, Unit for Epidemiology and Global Health, Umeå University, Umeå, Sweden; 2 Department of Clinical Sciences, Child and Adolescent Psychiatry, Umeå University, Umeå, Sweden; 3 Senior Lecturer, Department of Learning, Informatics, Management and Ethics, Medical Management Center, Karolinska Institutet, Stockholm, Sweden; 4 Senior Lecturer, Department of Public Health and Clinical Medicine, Epidemiology and Global Health, Umeå University, Umeå, Sweden; 5 Associate Professor/District Physician, Center for Family and Community Stockholm County Council, Karolinska Institutet, Stockholm, Sweden; 6 Professor of Social and Behavioral Sciences, Department of Learning, Informatics, Management and Ethics, Medical Management Center, KarolinskaInstitutet, Stockholm, Sweden

**Keywords:** clinical practice guidelines, depression, implementation, primary health care, randomized clinical trial

## Abstract

**Aim:**

The aim of this study is to investigate factors contributing to the failure of a randomized clinical trial designed to implement and test clinical practice guidelines for the treatment of depression in primary health care (PHC).

**Background:**

Although the occurrence of depression is increasing globally, many patients with depression do not receive optimal treatment. Clinical practice guidelines for the treatment of depression, which aim to establish evidence-based clinical practice in health care, are often underused and in need of operationalization in and adaptation to clinical praxis. This study explores a failed clinical trial designed to implement and test treatment of depression in PHC in Sweden.

**Method:**

Qualitative case study methodology was used. Semi-structured interviews were conducted with eight participants from the clinical trial researcher group and 11 health care professionals at five PHC units. Additionally, archival data (ie, documents, email correspondence, reports on the clinical trial) from the years 2007–2010 were analysed.

**Findings:**

The study identified barriers to the implementation of the clinical trial in the project characteristics, the medical professionals, the patients, and the social network, as well as in the organizational, economic and political context. The project increased staff workload and created tension as the PHC culture and the research activities clashed (eg, because of the systematic use of questionnaires and changes in scheduling and planning of patient visits). Furthermore, there was a perception that the PHC units’ management did not sufficiently support the project and that the project lacked basic incentives for reaching a sustainable resolution. Despite efforts by the project managers to enhance and support implementation of the innovation, they were unable to overcome these barriers. The study illustrates the complexity and barriers of performing clinical trials in the PHC.

## Introduction

The incidence and prevalence of depression is increasing in Sweden as well as in other western countries [World Health Organization (WHO), 2004; National Board of Health and Welfare (NBHW), 2013]. The burden of depression for individuals and their families is substantial. Depression is associated with morbidity and mortality (ie, suicide and cardio- and cerebro-vascular diseases) (Lépine and Briley, 2011). At some point in their lives, about one-third of the women and one-fourth of the men in Sweden experience depression severe enough to require treatment (NBHW, 2013). The majority of patients who suffers from depression are treated at primary health care units (PHC) [Swedish Council on Health Technology Assessment (SBU), 2004].

Several studies indicate that patients with depression do not receive the best treatment recommended by available knowledge (The WHO World Mental Health Survey Consortium, 2004; Pilling, 2008; NBHW, 2013). A recent report by the NBHW in Sweden concluded that the national guidelines for treating depression (introduced in 2009) were applied only to a very limited extent in Swedish clinical practice (NBHW, 2013). Of the ten PHC units the Board examined, only one unit reported that the national guidelines had resulted in practical changes such as greater resources for psychological treatment and improved psychiatric diagnostics.

In PHC, antidepressant medications, especially selective serotonin reuptake inhibitors (SSRIs), are most commonly used to treat depressive disorders. Although the use of antidepressant medication is convenient and often effective, there is evidence that psychological, or psychotherapeutic, treatment can also be effective (Cuijpers *et al.*, 2008). It is also well known that many patients, who are reluctant to take medication for problems they perceive as psycho-social and relational, prefer to talk to medical professionals (Dwight-Johnson *et al.*, 2000). Such professional counselling is typically unavailable in Swedish PHC units. Meeting the clinical guidelines for depression in Sweden would require a large change in the current treatment of depression.

Clinical practice guidelines (CPG) are ‘statements that include recommendations intended to optimize patient care. They are informed by a systematic review of evidence and an assessment of the benefits and harms of alternative care options’ [Institute of Medicine (IOM), 2011: 25]. In Sweden, the SBU and the NBHW produce national guidelines for health care. In 2004 a systematic review report ‘Treatment of Depression’ (CPG-D) summarized the evidence on treatments for depression (SBU, 2004). The CPG-D concluded that for treatment of mild and moderate depression, psychotherapy was as effective as tricyclic antidepressant (high quality of evidence) and probably as effective as SSRIs (moderate quality of evidence) (see Bowers, 1990). The launch of CPG-D was a national attempt to improve the treatment of patients with depression.

The difficulties encountered in implementing innovations and in using evidence-based guidelines in health care settings are well known (Grol *et al.*, 1998; Flottorp *et al.*, 2003; Brusamento *et al.*, 2012). Theoretical models have highlighted the influence of medical professionals’ perceptions and attitudes (Cabana *et al.*, 1999), the potential of various implementation strategies (Grimshaw *et al.*, 2006) and the significance of contextual organizational and political factors on adherence to CPGs (Green *et al.*, 1988; Greenhalgh *et al.*, 2004). Implementation of CPGs in PHC settings has been described as especially challenging (McKenna *et al.*, 2004; Rashidian *et al.*, 2008; Carlfjord *et al.*, 2010).

A systematic review of the effectiveness of strategies to implement CPGs (ie, for treatment of chronic diseases) at the primary care level in the European Union concluded that 19% of the studies reported fully effective implementation, 38% partially effective implementation and 43% no effect. Brusamento *et al.* (2012) found that the major implementation barrier to the use of CPGs in PHC was the lack of awareness and agreement about them. Internal factors such as organizational changes, staff shortages, inadequate time, resources and support were significant factors that inhibited implementation of CPGs in PHC units (Flottorp *et al.*, 2003; Carlfjord *et al.*, 2010). Grol’s (2001) evaluation of the implementation of 70 CPGs in PHC in the Netherlands showed that the implementation process benefitted from a thorough initial analysis of the target group and the target setting, as well as of the existing development structures in the organization.

In their examination of the driving forces for changes in health care praxis, Grol and Wensing (2004: 59) recommended an integrative, multilevel approach. In its assimilation of other empirical perspectives, their approach implies that incentives for and barriers to change derive from a number of sources. They list the following as barriers that are commonly found in complex health care settings: the characteristics of the innovation (the relative advantages, credibility, accessibility, attractiveness of the innovation); the involved professionals (their awareness, knowledge, attitude, motivation for change, behavioural routines); the patients (their knowledge, skills, attitude, compliance); the social network (culture created by colleagues’ opinions collaboration, leadership); the organization (care processes, staff, capacities, resources, structures); and the economic and political context (financial arrangements, regulations, policies). Grol and Wensing’s (2004) theoretical framework was used in this study to structure the barriers of performing a clinical trial.

### ‘Treating Depression in Primary care’: the DIP project

With the aim of investigating how evidence for treating depression – as formulated in the CPG-D – could be adopted and applied in PHC, a group of researchers and clinicians (the DIP research group) used the key recommendations of the CPG-D in the design of a randomized clinical trial (the DIP project). The group recognized practical and financial constraints that hindered the direct application of the CPG-D recommendations to PHC practice. DIP aimed to explore how the different treatment recommendations could be adapted to the PHC reality in a patient–physician–therapist setting and if the treatment effects would tolerate such an adoption. For evaluating the effects DIP used the randomized clinical trial (RCT)-design.

The project included three groups with different roles: (a) the DIP research group (*n*=10) who initiated, designed and directed the project and consisted of researchers, facilitators with specialization in clinical trials and clinicians with specialization in psychotherapy and psychiatry; (b) Educators (*n*=3), that were specialists in psychotherapy who taught and supervised the PHC clinicians in the psychological treatment method used in the project; (c) PHC staff members (*n*=20), consisting of PHC unit managers, screeners (GPs who collected data and followed patients in the project) and counsellors who were PHC clinicians (eg, GPs and nurses).

The DIP project consisted of the following interventions (summarized):

Preparing the clinical trial

∙ Development of a treatment manual of mild/moderate depression in a PHC setting based on the CPG-D recommendations (see Lindgren, 2007, for a more detailed description).
∙ A pilot trial at one PHC unit. Evaluations indicated positive treatment outcomes.
∙ Inclusion of five PHC units of different sizes in the Stockholm region in Sweden volunteering to participate in the clinical trial (reg. protocol no. 2007-001450-66).
∙ The PHC units received 2000 SEK (c. 220 €) for each patient in the project and 8000 Swedish crowns (c. 875 €) for each patient who completed the treatment procedure.
∙ A pharmaceutical firm financed the project (see Acknowledgements).

Support for PHC staff

∙ Education and continuously supervision to the Counsellors concerning the psychological treatment method.
∙ Distribution of educational materials (eg, check lists, patient information, counselling manual).
∙ Clinical research support from an independent organization (Karolinska Trial Alliance) during the process.
∙ Intermittent out-reach visits of a researcher-nurse to facilitate enrolment of patients.
∙ Two interactive workshops aiming to support the process and to identify barriers.
∙ Computerized systems to support structured decision-making surrounding the patient data collection at the PHC units.

Patient screening and inclusion

∙ Patients were screened on randomly chosen days by a researcher-nurse (MADRS-S self-rating depression scale, see Montgomery and Åsberg, 1979).
∙ Patients with mild or moderate depression (MADRS-S >12⩽20) and who volunteered for the project were included.

Treatment

∙ Randomization of patients to (a) the SSRI treatment method or (b) the psychological treatment method.
∙ The psychological treatment method was provided by the PHC unit’s trained staff (eg, GPs, nurses).

Follow-up

∙ GPs followed-up patients in the project on six occasions as required by the project’s trial protocol.
∙ Research data were collected from patient records, seven assessment questionnaires and various blood samples.

The DIP research group needed to enrol at least 240 patients in the project that fulfilled the inclusion criteria of mild or moderate depression. After 28 months, only 30 patients had enrolled in the project and the DIP research group ended the project. As targets were not reached the research group defined the early closure as a project failure and this view was shared by other participants.

Failed clinical trials are seldom published even though such results could contribute with crucial knowledge. Unsuccessful change interventions often meet the same fate. In contrast, this article presents conclusions from a failed clinical trial in PHC. The aim of this study is to investigate factors contributing to the failure of a RCT designed to implement and test CPG for the treatment of depression.

## Method

Using a qualitative case study approach (Yin, 2009), semi-structured interviews were conducted with eight participants in the clinical trial and with 11 health professionals at the PHC units. Archival data with historical and contemporary information relevant to the research project were also collected.

### Data collection

Interviews with five of the ten members of the DIP research group (C.S. was excluded due to his active role in the case study), all three educators and 11 of the 20 participating health professionals at the five PHC units (Table 1) were conducted. All active participants in the DIP project were invited to participate, but some declined or were not able to participate for other reasons (change of work place, etc.). The interviews were conducted at the five PHC units in the Stockholm region of Sweden and at the university that hosted the main activities of the DIP research group. We conducted 14 individual interviews and one group interview with five counsellors.

**Table 1:** Interviews of participants of the DIP project

Group	Semi-structured interviews	Group interview	∑
(a) DIP research group (*n*)	5		5
(b) Educators (*n*)	3		3
(c) PHC staff (*n*)	6	5	11
Total	14	5	19

DIP=depression in primary care; PHC=primary health care.

We used a semi-structured interview guide that focused on the aims, processes and outcomes of the project and on the factors that facilitated or hindered its implementation. Examples of interview requests/questions: ‘Describe the DIP project’s development and process from the beginning until the end’; ‘In your opinion, what influenced the early closure of the DIP project?’ These interviews, which lasted from 45 to 80 min, were recorded and transcribed verbatim. We allowed the respondents to read the transcripts and to suggest corrections if needed.

Additionally archival data (ie, email correspondence, reports on the clinical trial) from the years 2007–2010 were gathered. Archival data included 82 pages and described the history, context, purpose and process of the trial. Archival data describing the factors contributing to the failure of the clinical trial was included in the data analysis.

### Data analysis

We applied qualitative content analysis to the interview and parts of the archival data using a systematic classification process (Graneheim and Lundman, 2004). The classification process produced categories and sub-categories that sorted information about the latent and manifest content of the data.

The analysis was conducted in seven steps. First, the archival data was sorted chronologically. Sections of the reports and the emails that could relate to the failure of the clinical trial were selected for the further analysis. Second, we read the transcriptions from interviews and archival data several times to obtain a sense of the whole. Third, we coded the factors that hindered the implementation and realization of the projects’ aims (eg, descriptions of barriers, challenges and other difficulties). Fourth, we labelled those factors using a time code that indicated the period or periods during the DIP project when respondents described an obstacle. Fifth, we coded related words and sentences. Codes with similar content were divided into categories and sub-categories. Sixth, we compared and validated these categories and sub-categories with the transcribed interviews. Seventh, we placed the categories and sub-categories in a theoretical framework inspired by Grol and Wensing (2004). This framework suggests that incentives and barriers to change in health care are expected at various levels including the innovation, the professionals, the patients, the social context, the organization and the economic and political context. By innovation we refer to the DIP-project.

The first author (L.S.) conducted the interviews, performed the preliminary coding and coordinated the sessions with two authors (C.S. and M.N.) in order to confirm the trustworthiness of the research findings. Two authors (L.S. and C.S.) then discussed and reviewed the categories.

## Results

Several factors influenced the failed effort to perform the clinical trial based on the CPG-D recommendations. Table 2 presents an overview of these implementation barriers. Statements by interview respondents are used to exemplify and clarify themes, categories and sub-categories.

**Table 2:** Overview of themes, categories and sub-categories of implementation barriers in the DIP project according to the Grol and Wensing (2004) framework

	Theme	Sub-category level 1	Sub-category level 2
The innovation	Project purpose	Difficulties in realizing the purposes	RCT-study, patient enrolment, data collection failed
		of the DIP project	Development of treatment of depression in PHC units partially failed
			Work environment improvements failed
	Design	RCTs are extensive and complex	Extensive methodology
			Difficult to fulfil in a clinical context
			Strains among DIP components (eg, screening, pharmacological treatment and psychological treatment)
			Questionnaires were time consuming and impractical
	Content	Tensions between DIP Project components	Different components of the project were valued differently (eg, screening, pharmacological treatment and psychological treatment)
		Screening procedure challenging patients’ rights	Waiting room screening exposed patients, endangering patient confidentiality
			Unethical to approach patients about their psychiatric status when they were at the PHC for other reasons
		Treatment procedures that challenged	Challenging professional roles and behaviours
		the professional roles and PHC routines	Challenging PHC routines concerning length/frequency of patient visits
			Challenging terminology and principles of patient interviews/counselling
	Implementation	Stress associated with implementation	Meetings for education, supervision and/or training time-consuming
	activities	activities	Visits from DIP research group were time-consuming
			Conflicts emerged during out-reach visits
			Reminders and feedback system contributed to stress
The professionals	Attitudes/Motivation	Underestimation of project resources	More resources needed in terms of own work than predicted/initially described
		DIP deteriorating work environment	DIP increased work load and/or stress
		Lack of agreement with the CPG-D	CPG-D viewed as unrealistic
			New CPG-Ds unnecessary, previous agreements about treatment are valid
			SSRI exclusive use is effective and a better treatment for the patient group
		Lack of agreement with the DIP project	Negative attitudes towards PHC medical staff performing psychotherapy
			Experiences of not performing according to contract
	Behaviour	Not participating actively in project	Not including patients in the study
			Not attending DIP meetings
			Deficient communications between participants
The patients	Negative attitudes	Towards depression	Patients not wanting to accept their own depression/mental illness
		Towards screening procedure	Negative experiences of being faced with unexpected questions about mental health at PHC visit
			Negative experiences of being overtly approached in waiting room
	Patient	CPG-D/DIP did not suit the	PHC patients having multiple complex disorders, CPG-D too rigid
	characteristics	patient group	Most patients with mild or moderate depression were known
		Few patients	New cases of depression did not occur at the expected pace
The social network	Collaboration DIP	Participants’ needs not considered in	Meetings not responding to PHC needs (location, topics, language)
	network and PHC	meetings	Experiences of declining interest in participating in meetings
	Cultures in the network	Clash between research and clinical praxis	PHC staff experienced DIP language and procedures/methods as foreign and separated from PHC practices
			Experienced difficulties and limited option to perform research in PHC
The organizational context	Leadership	Ambivalent leadership in PHC	Ambivalence about participating in and realizing the aims of the DIP Leaders unclear in communications and unavailable in meetings PHC management charged with complex, multifaceted tasks
		Ineffective decision making process in PHC participating in DIP	Insufficient anchoring of DIP project with PHC management Insufficient anchoring of DIP with PHC GPs and nurses by PHC management Incomplete analysis of the resource implications for PHC units in the DIP
	Capacities/resources	Staff turnovers in PHC and in DIP research group	High degree of staff turnovers in PHC during the research period
	Terms in PHC	Strenuous work environment in PHC	Work stress in clinical work, at the PHC units
			Stress and insecurity in PHC units due to fundamental organizational changes
			Low control in PHC work situation and unpredictable workloads
	Organizational	Shortage of staff – low priority for	Development projects not prioritized when staff shortage occurred
	structures	Research and Development (R&D)	Temporary GPs not suited/allowed to participate in DIP
		activities	Strict prioritizations of available GPs work tasks
		Coinciding organizational changes in PHC – low priority for R&D	‘Freedom of Choice in Health Act’ reform demanded time and resources
			‘Freedom of Choice in Health Act’ reform redefining PHC’s core task
			Temporary emergency organization for large-scale swine-flu vaccinations
The economic and political context	Financial arrangements	R&D not one of PHC core tasks	Changed conditions for finical reimbursement, gratifying accessibility
	Regulations Policies		PHC changed ownership from public to private

DIP=depression in primary care; RCT=randomized clinical trial; PHC=primary health care; CPG-D=clinical practice guideline depression; SSRI=selective serotonin reuptake inhibitor.

### The innovation: the DIP project

The main goal of the DIP project, as described in both formal steering documents and in interviews, was two-fold: to conduct a clinical trial and to advance depression treatment according to current guidelines. Participants from all groups in the DIP project had additional goals and expected still other results from the project (eg, stimulation of the research tradition within the PHC units, work environment gains and career promoting opportunities for the GPs). To some degree, the Counsellors at the PHC units found that these additional goals and expectations were met. Other staff members at the PHC units disagreed, mainly because of the increased work stress. Members of the DIP research group realized that the project goal of stimulating research in PHC was rather unattainable. These results were offered as explanations for their decreased involvement in the DIP project.

‘I guess the expectation I had for the project was unrealistic. Because I thought it would decrease my workload, initially it felt like a fresh breath of air. But eventually it just added to other work burdens’. (PHC manager)

The DIP project design, a randomized, controlled clinical trial, was viewed by the majority of the respondents as a barrier to implementation of the CPG-D recommendations. Some respondents claimed the project design involved an extensive methodology that increased the complexity of the project artefacts (ie, the protocols, instructions and manuals) and activities. The design also contributed to a view of the DIP project as a foreign project, alien to the PHC culture. Other respondents thought that the project design was too demanding and inflexible because it required controlled, systematic activities in order to achieve its research objectives. Moreover, some respondents thought that the repeated use of structured questionnaires was too time-consuming and too difficult to integrate with the patient visits.

The Counsellors and the Educators observed that the psychological treatment method presented a challenge to professional roles and behaviours. As a result, new and different work tasks, procedures and skills were needed. Interviews and archival data express that the psychological treatment method, which demanded changes in the scheduling and planning of patient visits, had administrative implications for the PHC units.

‘I usually dictate the journal directly in the room with the patient present. It helps me to keep up with the time schedule. But it is not appropriate in this kind of [patient] meeting’. (Nurse, Counsellor)

Some Counsellors also declared that the PHC units’ facilities layout and equipment were not suitable for counselling sessions.

‘As you can see [pointing to a tray with medical instruments and to a desk stacked with files], these rooms do not say to the patient, “Sit down and tell me how you really feel”. And it does not matter if the light outside my door is red, indicating do not disturb. People will push open the door and rush in anyway. I have to lock it!’ (Nurse, Counsellor)

The PHC staff members also thought it was difficult to find time to perform activities (eg, training, seminars, out-reach visits and reminders) given their normal working schedules.

### The professionals

**Figure 1:**
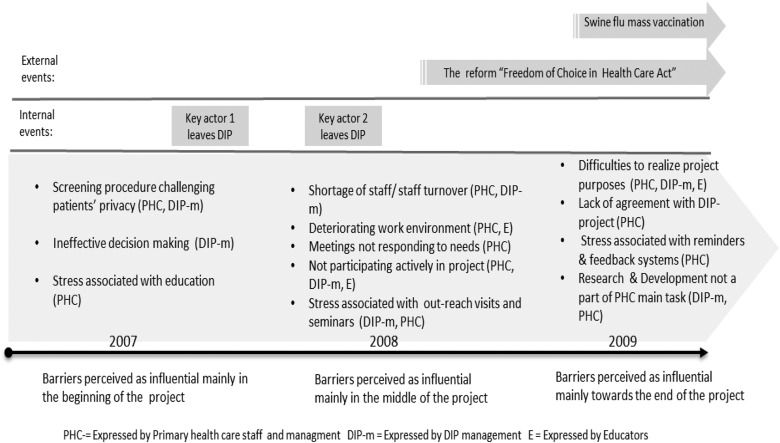
Chronology of key events and barriers in the DIP project. PHC=expressed by primary health care staff and management; DIP=depression in primary care; DIP-m=expressed by DIP management; E=expressed by Educators.

Some respondents linked their negative responses to the DIP project to the increased work load. The PHC staff members and parts of the DIP research group stated they had underestimated the amount of time and effort the project demanded. Some of them described how these negative responses were reinforced when other internal or external events occurred and were prioritized within the PHC unit (Figure 1). Some respondents said that this was the mental turning point when they decided to leave the project.

‘And then the swine flu vaccination came. That was the last straw for me. I thought then, “I can’t do all of these things”. And because the vaccination programme was a top priority in PHC, I thought to myself, “I can’t do DIP anymore”. Even though I really wanted to and believed in it.’ (GP, Screener)

Some PHC staff members and DIP research group members described how they withdrew from the DIP project activities. For example, they declined to participate in project implementation activities, decreased their project communications, ceased using the questionnaires according to the protocols and/or stopped the enrolment of patients.

A few respondents thought the negative attitudes towards the CPG-D recommendations were related to doubts about the accuracy of the underlying medical evidence. These respondents evaluated other treatment recommendations for depression (ie, pharmacological treatment) as more effective. There was also concern that the DIP project, which was premised on the idea that PHC medical staff could also conduct psychotherapy, promoted economic solutions over medical solutions.

### The patients

The interviews showed that the staff members at the PHC units attributed some of the failure of the DIP project to various patient-related factors. Among these factors, which accounted for low enrolment in the project, were patient characteristics, attitudes and experiences related to the treatment of depression. Some respondents argued that the number of patients who met the inclusion criteria was low in the population that sought care at PHC units. Furthermore, they discovered some patients were unwilling to admit to symptoms of depression or were uncomfortable with the waiting room approach in which questions were asked about their mental health. In addition, respondents thought it was unethical to ask patients mental health questions when the patients were seeking other health care treatment. Such screening and questionnaire procedures were perceived to potentially undermine patient confidentiality and/or interfere with the physician/patient relationship. Some PHC staff members also stated that because PHC patients often have multiple and complex disorders, the treatment recommendations, featured in the DIP project, were inflexible and not appropriate for the patient group.

### The social network

A majority of respondents described the cultural clash between the research tradition and the PHC tradition. As a result, the PHC units were reluctant to participate in the research. In part, communication difficulties between the researchers and the PHC staff members caused this clash.

‘There is no tradition of research in primary care. We have no experience and are almost afraid of projects such as this [although] there is a lot of respect for research as well. The project was like a large, strange bird in our organization. Our main task is to quickly respond and treat our patients’. (PHC Manager)

PHC staff members also thought the project meetings did not meet their needs in terms of location, topics and terminology use. Some respondents said the collaboration between the PHC management and the PHC staff members negatively influenced the project. They pointed to the low level of trust between leaders and co-workers. A few respondents stated that the PHC staff did not follow the PHC managements’ instructions about participation in the project because of their heavy workload and/or low interest. As a result, it was difficult to manage some professional groups in the project.

### The organizational context

Respondents identified four factors in the organizational context that contributed to the project failure: ambivalent leadership, high staff turnover, poor work conditions and conflicting organizational changes. A majority of the participants thought the PHC leadership of the DIP project at the PHC units was ineffective as far as the project’s planning, execution and outcomes. Various respondents from all groups claimed that their leaders (the PHC management and the DIP research group) failed to anchor DIP in the PHC units. Several saw this deficiency as a significant barrier to the project’s long-term survival. Some respondents complained that leaders could not realistically describe and/or assess what the project involved or demanded.

Several respondents from the PHC units and a few from the DIP research group changed work sites or functions during the project period. This resulted in staff changes and shortages in some cases and also meant that information was lost and project participation declined. Additionally, staff shortages in the PHC units in general meant that in the subsequent ordering of work tasks in research and development projects like DIP project were not prioritized.

Some respondents from the PHC units described how the work stress affected their participation. They were too exhausted to communicate, to learn new skills and/or to experience satisfaction in developing their practices. At the DIP seminars and kick-off meetings, when they felt invigorated, they agreed to participate in the project. However, when they returned to work, this enthusiasm waned as they tried to cope with the increased workload.

‘When we were at the project’s kick-off meeting, I felt that the atmosphere was good. And I thought it would work out. I wanted to participate. We need to improve our treatment of patients with depression. But then when I returned to our unit, the workload became too high and my motivation for the project declined’. (GP, Screener)

Some major events occurred during the course of the DIP project, revealed in archival data. See Figure 1 for a summary of events perceived as significant. One significant event was the national health care law ‘Freedom of Choice in Health Care’ that came into force in January of 2009. Respondents declared that the law implied a major organizational change at the PHC units. The units’ priority now was to improve patient access and to increase the number of patient visits. Participants thought that because the PHC units were occupied with this change, their motivation to participate in research and development projects, including the DIP project, decreased as a result of this reform.

‘During this time when the “Freedom of Choice in Health Care” reform was launched, everything was up-side-down. We did not know if we would even exist as a PHC unit when the year was over. Of course this affected the execution of DIP’. (PHC Manager)

Another major event was the national vaccination programme following the swine flu outbreak in 2010. The PHC units had responsibility for administering the vaccinations, and for a period of time this programme received high priority and the focus on the DIP project naturally decreased.

### The economic and political context

Several PHC staff members and managers perceived that the PHC units’ main mission and economic incentives had changed during the years that the DIP project was active. In particular, they pointed to the Swedish health care financial model that bases its reimbursements on the number of patient-visits at each PHC unit and to the introduction of the ‘Freedom of Choice in Health Care’ law. This change of focus in the political and economic management of health care was highlighted as a major barrier to the DIP implementation by a majority of the participants. Because the PHC units’ main mission did not include research and/or development, the conclusion was that the DIP project was not a part of their mission.

‘The overall aim in primary health care has evolved into being constantly available for all patients. I understood that this focus decreased their [the staff members’] motivation to participate in our project. The managers’ motivation declined when they realized that the reform “Care Choice Stockholm” contained no specific focus on research and development initiatives’. (Researcher, DIP research group)

‘The new financial system for primary health care is not compatible with the implementation of new clinical guidelines or research and development activities. It exclusively rewards according to the number of patient-visits’. (GP, Screener)

## Discussion

The DIP project was an effort to adopt and evaluate an operationalization of guidelines for treating depression in a clinical trial that closed early due to difficulties to enrol patients. In this case study we explore the factors that hindered the clinical trial in the PHC setting. The project demanded resources, efforts (eg, time, involvement, interest) and it implied change. New strict routines and practices that needed to co-exist with the prevailing conditions of PHC (eg, high workload, staff shortage) and also with other change processes and unexpected events (eg, large-scale health care reform, mass vaccinations). This is the reality that implementation efforts of any kind face in complex health care settings as PHC and represent the need for tailored interventions (eg, Baker *et al.*, 2010) and adherence to the influence of context on implementation and development (eg, Dopson *et al.*, 2008; Kaplan *et al.*, 2010; 2012; Bate, 2014).

The medical professionals eventually resisted the DIP project despite their initial positive attitudes towards it. The reasons were primarily the organizational changes, increased workloads, communication problems and the cultural clashes between clinical praxis and research. Still some barriers seem to have had a more superior role – the lack of leadership and incompatibility with the prevailing way of organizing and delivering care.

Initial motivation to participate declined as chains of problems arose; trying to cope with these problematic events the lack of anchoring, incitements and the diminished prioritization of the project were revealed. The lack of leadership support for the DIP project may have been the single most important barrier to its success. That key actors, such as managers, that influence change has a long-term commitment, provide support and enhance sense-making is an important factor when trying to improve the quality of services in a health care organization (Nyström, 2009).

The concept of compatibility is a significant factor that explains the problem of conducting research in the PHC setting (Carlfjord *et al.*, 2010). The perception of mismatch – that was most often connected to the research features of the DIP project – was a significant impediment to the implementation process. In Carlfjord *et al.*’s study impeding factors found were organizational changes and staff shortages that coincided with the implementation of a computer-based test for lifestyle in PHC settings, similar to the barriers found in this study. Our results are also in line with findings from a trial to implement guidelines in 120 general practices in Norway that concluded that lack of time, resources and support were the most salient factors that could explain the low uptake of guidelines (Flottorp *et al.*, 2003).

The theoretical framework used (Grol and Wensing, 2004) was useful for structuring the barriers. The purpose of identifying barriers to implementation on different levels is to be able to address them when planning for and executing complex change processes in health care. Many similar frameworks exist and a recent review proposed a comprised integrated checklist of determinants of practice (the TICD checklist) with seven domains and 57 determinants (Flottorp *et al.*, 2013). A missing part of both the used framework and TICD is the time and process factor, illustrated in Figure 1. Barriers interacted and were influenced by the key events that occurred during the DIP process. This result suggests a need to continuously monitor barriers during an implementation process and future frameworks or taxonomies could benefit from addressing the time and process factor.

The use of RCT as the gold standard of research has been questioned (eg, Grossman and Mackenzie, 2005; Cartwright, 2007; Hansson, 2014). In this case the RCT research design was present in two phases; the CPG-D was based mainly on RCT-studies and when adapting them to clinical practice with the help of the DIP-project the evaluation of the effect of the new approach used an RCT design. The case demonstrates that a methodology with high degree of control and systematics and a high scientific value may operate as an intimidating and to time-consuming procedure in a real health care setting. In other studies the challenges to perform RCTs on interventions in PHC have also been discussed, for example in relation to validity (Godwin *et al.*, 2003) or complexity of the intervention (Campbell *et al.*, 2000). Aiming to make use of evidence and science in clinical praxis one must ask how the different research paradigms can be used. When interventions are complex, maybe smaller pilot tests of new approaches, with room for interaction, adaptation and changes, could precede more robust designs aiming for test of causal relations?

Research and education are the missions of the universities and the university hospitals. Despite their differences in assignment, these groups interact. Using the best available knowledge universities train health professionals in clinical settings (ie, the PHC units) where health professionals can collect new clinical data. However, as this study illustrates, there are fundamental impediments in this exchange relationship. While many factors contributed to the failure of the DIP project, one important factor was likely the problem of uniting the missions of the two groups. It was too difficult to combine the PHC units’ primary activity (ie, meeting patients) and their core value (ie, patient access) with the activities (ie, medical data collection) and values (ie, the advancement/dissemination of knowledge) associated with the DIP project.

### Methodological considerations

In a retrospective study, methodological problems are a concern. Inaccuracies due to respondents’ recall errors may appear. In addition, there is the problem of author-researcher bias because the project outcome is known. However, the study focused on understanding the reasons for the outcome and the procedure for analysis is presented in detail. In this sense the study provides a reliable and detailed description of the implementation failure from the participants’ perspectives.

## Conclusion

This study shows the barriers of performing clinical trials in PHC and some of the complexities of adopting guideline recommendations and changing clinical practices in PHC settings. As it appears the production of health care and production of knowledge implied a conflict in work tasks, motivation and culture that was too difficult to bridge in this case.

In order to support the important collaboration and exchange between academia and health care, prerequisites for research in health care, and especially in distributed primary care units needs to be established. Clinical systems of documentation, routines, resources and physical settings needs to be prompted, aiming for a high quality health care ready to both treat and develop.
